# Temporospatial Expression of Fgfr1 and 2 During Lung Development, Homeostasis, and Regeneration

**DOI:** 10.3389/fphar.2020.00120

**Published:** 2020-03-02

**Authors:** Tingting Yuan, Kylie Klinkhammer, Handeng Lyu, Shan Gao, Jie Yuan, Seantel Hopkins, Jin-San Zhang, Stijn P. De Langhe

**Affiliations:** ^1^ Department of Medicine, Division of Pulmonary, Allergy & Critical Care Medicine, University of Alabama at Birmingham, Birmingham, AL, United States; ^2^ School of Pharmaceutical Sciences, Wenzhou Medical University, Wenzhou, China

**Keywords:** lung, Fgf, homeostasis, development, regeneration

## Abstract

Fgfr1 (Fibroblast growth factor receptor 1) and Fgfr2 are dynamically expressed during lung development, homeostasis, and regeneration. Our current analysis indicates that Fgfr2 is expressed in distal epithelial progenitors AT2, AT1, club, and basal cells but not in ciliated or neuroendocrine cells during lung development and homeostasis. However, after injury, Fgfr2 becomes upregulated in neuroendocrine cells and distal club cells. Epithelial Fgfr1 expression is minimal throughout lung development, homeostasis, and regeneration. We further find both Fgfr1 and Fgfr2 strongly expressed in cartilage progenitors and airway smooth muscle cells during lung development, whereas Fgfr1 but not Fgfr2 was expressed in lipofibroblasts and vascular smooth muscle cells. In the adult lung, Fgfr1 and Fgfr2 were mostly downregulated in smooth muscle cells but became upregulated after injury. Fgfr1 remained expressed in mesenchymal alveolar niche cells or lipofibroblasts with lower levels of expression in their descendant (alveolar) myofibroblasts during alveologenesis.

## Introduction

Fgfr1 and Fgfr2, two of the four fibroblast growth factor receptors, play important roles during lung development and regeneration, often mediating reciprocal signaling between the epithelium and mesenchyme *via* their ligand Fgfs (Jaskoll et al., 2005; MacKenzie et al., 2015; Balasooriya et al., 2017; Yuan et al., 2019). Our recent studies showed that Fgf10-Fgfr2b signaling is critical for generating basal cells and to drive alveolar epithelial regeneration after bleomycin injury in the lung (Yuan et al., 2019). Both Fgfr1 and Fgfr2 are also considered as potential targets for lung cancer therapy (Weiss et al., 2010; Theelen et al., 2016). However, most studies have focused on Fgfr1 or Fgfr2 signaling pathways. The temporospatial expression pattern of both Fgfr1 and Fgfr2 has not been carefully assessed.

In this study, using two mouse models featuring nuclear expression of cerulean under control of the *Fgfr1* promoter and nuclear expression of mCherry under control the *Fgfr2* promoter, we sought to explore the temporospatial expression pattern of these two receptors at the single cell level, during lung development, homeostasis and response to naphthalene or bleomycin injury. Since Fgf signaling regulates the expression of its receptor, this expression profile will not only provide a spatiotemporal map of which cells respond to Fgfr1 or Fgfr2 signaling at a given point in time, but also to what extent. Note that both Fgfr1 and Fgfr2 have two isoforms, b and c, which are respectively thought to be expressed in the epithelium vs mesenchyme; however, our reporter line cannot distinguish between these two isoforms.

### Fgfr1 and Fgfr2 Expression During Embryonic Lung Development

**Figure 1:**
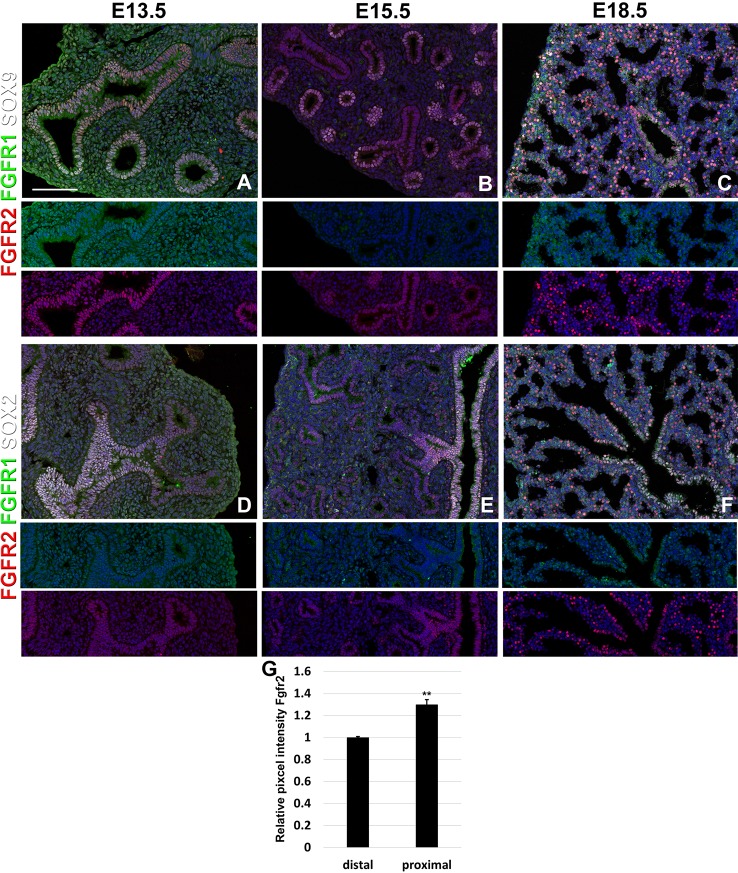
Epithelial Fgfr1 and Fgfr2 expression during embryonic lung development. **(A–F)** Immunostaining on E13.5, E15.5, and E18.5 *Fgfr1-Cerulean;Fgfr2-mCherry* lungs for GFP (Fgfr1-Cerulean, green), RFP (Fgfr2-mCherry, red), Sox9 (white), or Sox2 (white). Lower panels for each individual image show GFP (green) and RFP (red) channels only without the far red channels. **(G)** Quantification of relative average pixel intensity for Fgfr2 in distal vs proximal epithelium at E15.5. Data are mean ± s.e.m. **P < 0.01, as determined by a two-tailed t-test; n = 6 biological replicates for each experimental group. Scale bars, 100 µm.

**Figure 2:**
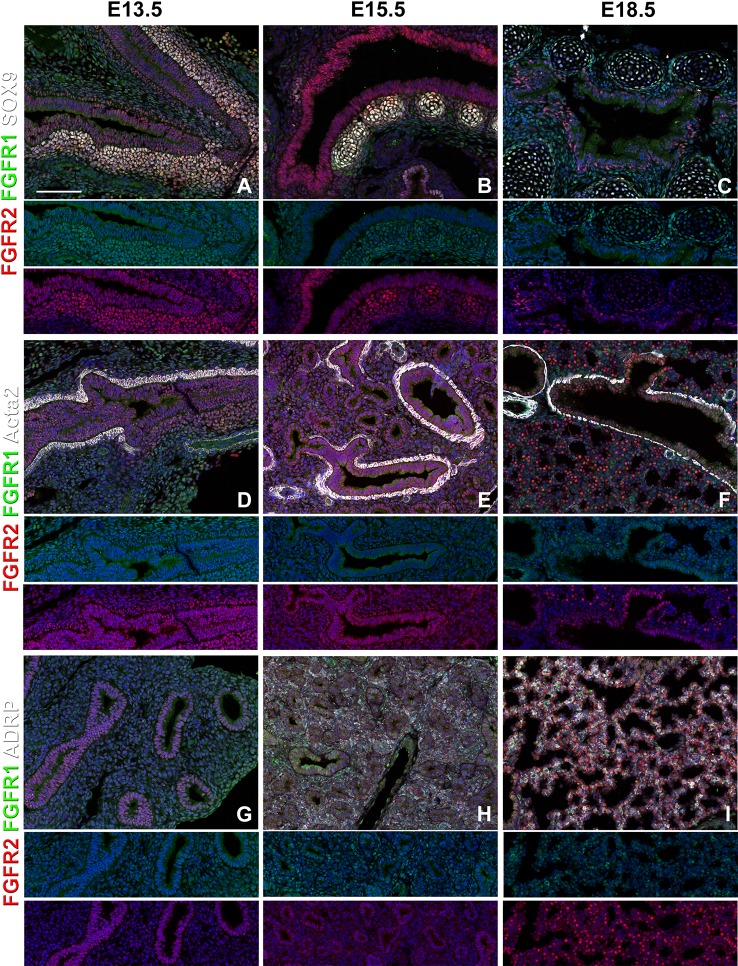
Mesenchymal Fgfr1 and Fgfr2 expression during embryonic lung development. **(A–C)** Immunostaining on E13.5, E15.5, and E18.5 *Fgfr1-Cerulean;Fgfr2-mCherry* proximal lungs for GFP (Fgfr1-Cerulean, green), RFP (Fgfr2-mCherry, red), Sox9 (white); **(D–I)** Immunostaining on E13.5, E15.5, and E18.5 *Fgfr1-Cerulean;Fgfr2-mCherry* lungs for GFP (Fgfr1-Cerulean, green), RFP (Fgfr2-mCherry, red), Acta2 (white), or ADRP (white). Lower panels for each individual image show GFP (green) and RFP (red) channels only without the far red channel. Scale bars, 100 µm, n ≥ 6.

We find that during early lung development, around E13.5, Fgfr1 is moderately expressed in both the epithelium (Sox9, Sox2) and mesenchyme (Acta2, Adrp) (**Figures 1A, D** and **2D, G**) and is upregulated in Sox9^+^ cartilage progenitors (**Figure 2A**). At E15.5, Fgfr1 is expressed at low levels in the epithelium and remains expressed in developing lipofibroblasts (**Figures 1B** and **2H**), vascular smooth muscle cells, and cartilage progenitors (**Figures 2B, E, H**). By E18.5, Fgfr1 expression becomes relatively restricted to ADRP^+^ lipofibroblasts aka mesenchymal AT2 niche cells (MANC) (**Figure 2I**), vascular smooth muscle cells (**Figure 2F**) and tracheal cartilage (**Figure 2C**), with low expression levels in the conducting airway (**Figures 1C, F**).

Interestingly, we found Fgfr2 expression to be homogenously expressed in the lung epithelium at E13.5 (**Figures 1A, D**) as well as in airway smooth muscle cells (**Figure 2D**), and Sox9^+^ cartilage progenitors (**Figure 2A**). At E15.5, Fgfr2 is expressed in the epithelium with higher levels proximally vs distally (**Figures 1B, E, G**), airway smooth muscle cells (**Figure 2E**), and cartilage progenitors (**Figure 2B**). A downregulation of Fgfr2 signaling distally around E15.5 is required for the lung to transition from a branching program into an alveolar differentiation program; as we have previously demonstrated that overexpression of *Fgf10* starting from E15.5 onwards prevents this transition by inducing distal epithelial Fgfr2 signaling (Volckaert et al., 2013b; Volckaert et al., 2019). Upregulation of Fgfr2 in the proximal epithelium at this stage also coincides with the differentiation of the basal cell lineage (Volckaert et al., 2013b; Balasooriya et al., 2017; Volckaert et al., 2017; Volckaert et al., 2019).

**Figure 3:**
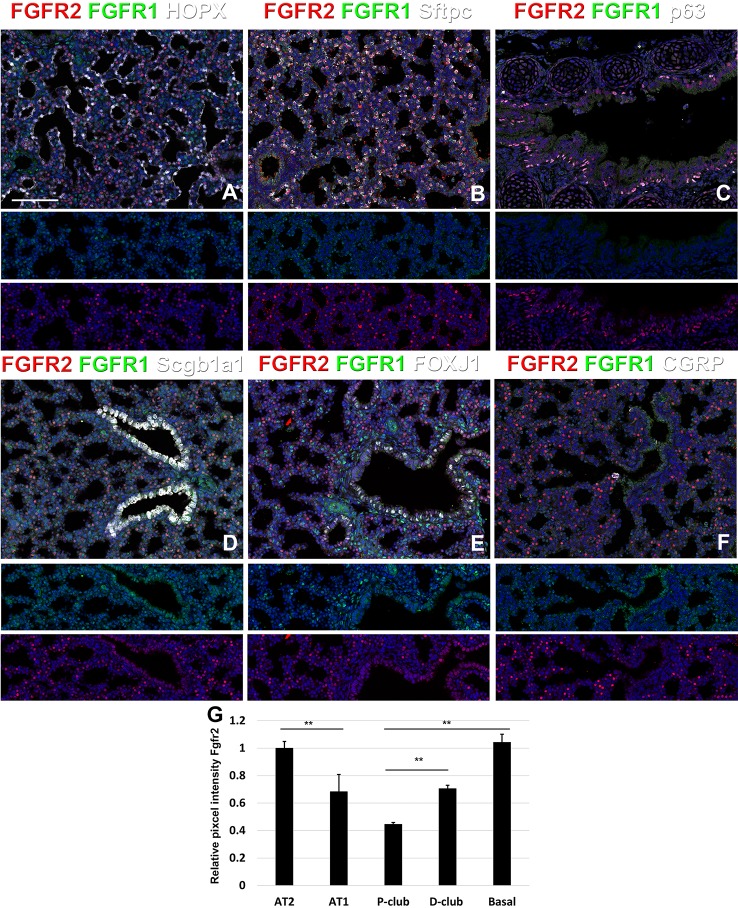
Fgfr1 and Fgfr2 expression during late embryonic lung development in specific epithelial lineages. **(A–E)** Immunostaining on E18.5 *Fgfr1-Cerulean;Fgfr2-mCherry* lungs for GFP (Fgfr1-Cerulean), RFP (Fgfr2-mCherry, red), Hopx (white), Sftpc (white), p63 (white), Scgb1a1 (white), Foxj1 (white), or CGRP (white). **(G)** Quantification of relative average pixel intensity for Fgfr2 in alveolar type 2 (AT2) cells, AT1 cells, proximal and distal club cells and tracheal basal cells at E18.5. Data are mean ± s.e.m. **P < 0.01, as determined by a two-tailed t-test; n = 6 biological replicates for each experimental group. Scale bars, 100 µm.

At E18.5 we find Fgfr2 to be highly expressed in the developing AT2 cells (**Figures 3B, G**), basal cells (**Figures 3C, G**), distal airway clubs at the BADJ (**Figures 1F** and **3G**) and in cartilage cells (**Figure 2C**), with lower levels of expression in AT1 (**Figures 3A, G**) and proximal club cells (**Figures 3D, G**) and surrounding airway smooth muscle cells (**Figure 2F**). Furthermore, we find that at E18.5, there is no Fgfr2 expression in Foxj1^+^ ciliated cells or CGRP^+^ neuroendocrine cells (**Figures 3E, F**). These observations are consistent with our previous reports that overexpression of *Fgf10* during late lung development blocks the differentiation of AT1 and ciliated cells in favor of AT2 and club or basal cells, respectively (Volckaert et al., 2013b; Volckaert et al., 2019).

### Fgfr1 and Fgfr2 Expression During Postnatal Lung Development

**Figure 4:**
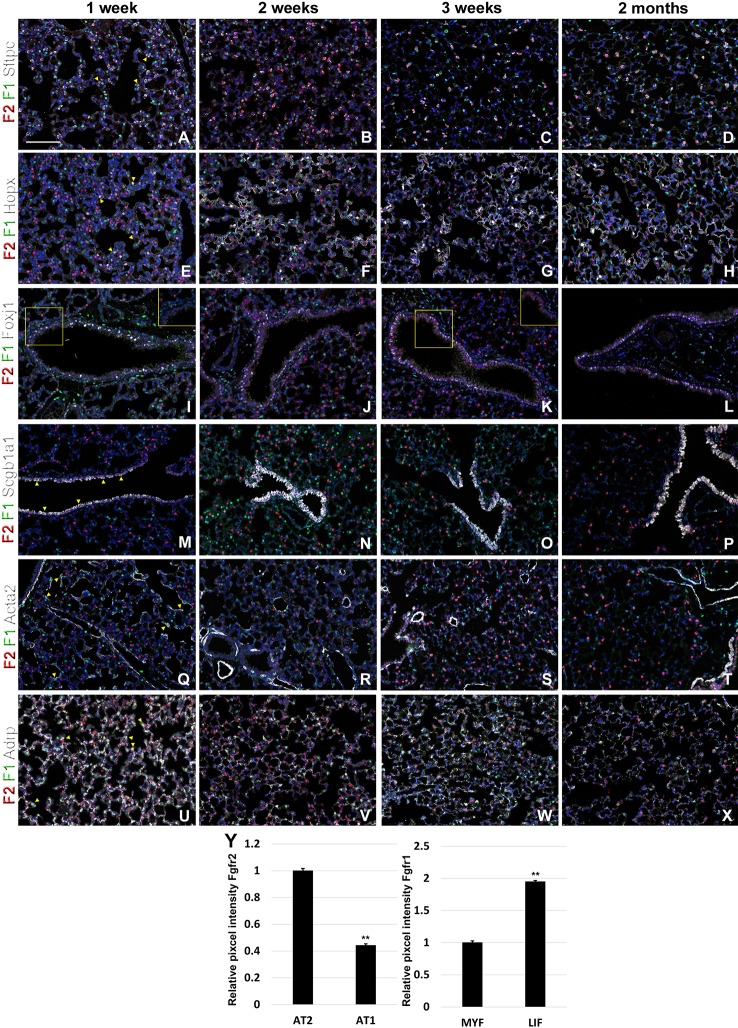
Fgfr1 and Fgfr2 expression during postnatal lung development. **(A–X)** Immunostaining on P7, P14, P21, and 2 month old *Fgfr1-Cerulean;Fgfr2-mCherry* lungs for GFP (Fgfr1-Cerulean, green), RFP (Fgfr2-mCherry, red), Sftpc (white), Hopx (white), Fox1 (white), Scgb1a1 (white), Acta2 (white), or ADRP (white) (arrowheads indicate double labeled cells whereas frames show only GFP and RFP). **(Y)** Quantification of relative average pixel intensity for Fgfr2 in alveolar type 2 (AT2) vs AT1 cells at 2 months of age or Fgfr1 in alveolar myofibroblasts vs lipofibroblasts at P7. Data are mean ± s.e.m. **P < 0.01, as determined by a two-tailed t-test; n ≥ 3 biological replicates for each experimental group. Scale bars, 100 µm.

During postnatal lung development, we found strong Fgfr2 expression in AT2 cells and strong Fgfr1 signaling in adjacent mesenchymal alveolar niche cells or lipofibroblasts (**Figures 4A–D, U–Y**). Interestingly, Fgfr2 expression was still present in AT1 cells but at lower level than in AT2 cells (**Figures 4E–H, Y**), which is consistent with an important role for Fgfr2 signaling in AT2 stem cell maintenance (Yuan et al., 2019). We further found Fgfr2 expression in club cells (**Figures 4M–P**), but not in ciliated cells (**Figures 4I–L**). Expression of Fgfr1 and 2 was low in airway and vascular smooth muscle cells (**Figures 4Q–T**). However, alveolar myofibroblasts exhibited modest Fgfr1 expression at P7 and P14 (**Figures 4Q, R, Y**), which is consistent with a lineage relationship with lipofibroblasts (Al Alam et al., 2015; El Agha et al., 2017; Li et al., 2018).

### Dynamic Fgfr1 and Fgfr2 Expression After Naphthalene or Bleomycin Injury

**Figure 5:**
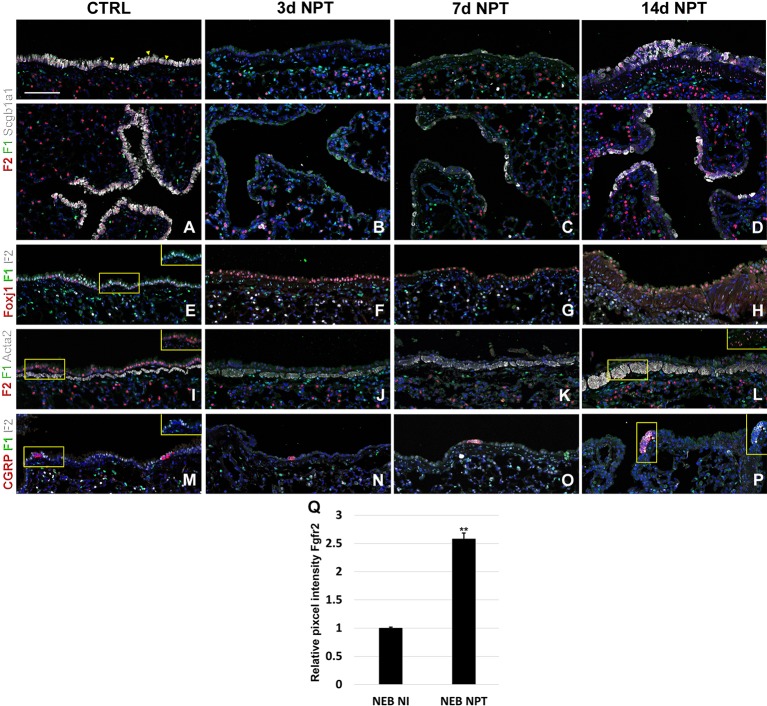
Fgfr1 and Fgfr2 expression in the adult lung after naphthalene injury. **(A–P)** Immunostaining on ctrl and naphthalene injured adult *Fgfr1-Cerulean;Fgfr2-mCherry* lungs for GFP (Fgfr1-Cerulean, green), RFP (Fgfr2-mCherry, red), Scgb1a1 (white), Foxj1 (white), Acta2 (white), or CGRP (white) (arrowheads indicate double labeled cells whereas frames show only GFP and RFP). **(Q)** Quantification of relative average pixel intensity for Fgfr2 in NEBs after naphthalene injury. Data are mean ± s.e.m. **P < 0.01, as determined by a two-tailed t-test; n ≥ 5 biological replicates for each experimental group. Scale bars, 100 µm.

Next, to investigate the dynamic changes in both Fgfr1 and Fgfr2 expression after lung injury, we performed naphthalene and bleomycin injuries on adult mice at 8 weeks of age and traced the expression changes during lung regeneration. As expected, we found that Fgfr2 expression was pretty much gone in the airway at 3 and 7 days after naphthalene injury, consistent with a loss of club cells the main Fgfr2 expression cell type in the adult conducting airway (**Figures 5A–C**, **E–G** and **S1**) (Volckaert et al., 2011). However, upon the return of club cells, Fgfr2 expression was gradually restored by 14 days after naphthalene injury (**Figures 5D**, **H**). We further found a modest increase in both Fgfr1 and Fgfr2 signaling in the airway smooth muscle cells upon naphthalene injury, consistent with an activation of this stem cell niche upon injury (**Figures 5I–L**) (Volckaert et al., 2011; Volckaert et al., 2013a; Lee et al., 2017). Remarkably, we found Fgfr2 upregulated in neuroendocrine bodies upon naphthalene injury, consistent with a role for Fgf10 signaling in regeneration of the airway epithelium by this distinct stem cell population (**Figures 5M–Q**) (Volckaert et al., 2011).

**Figure 6:**
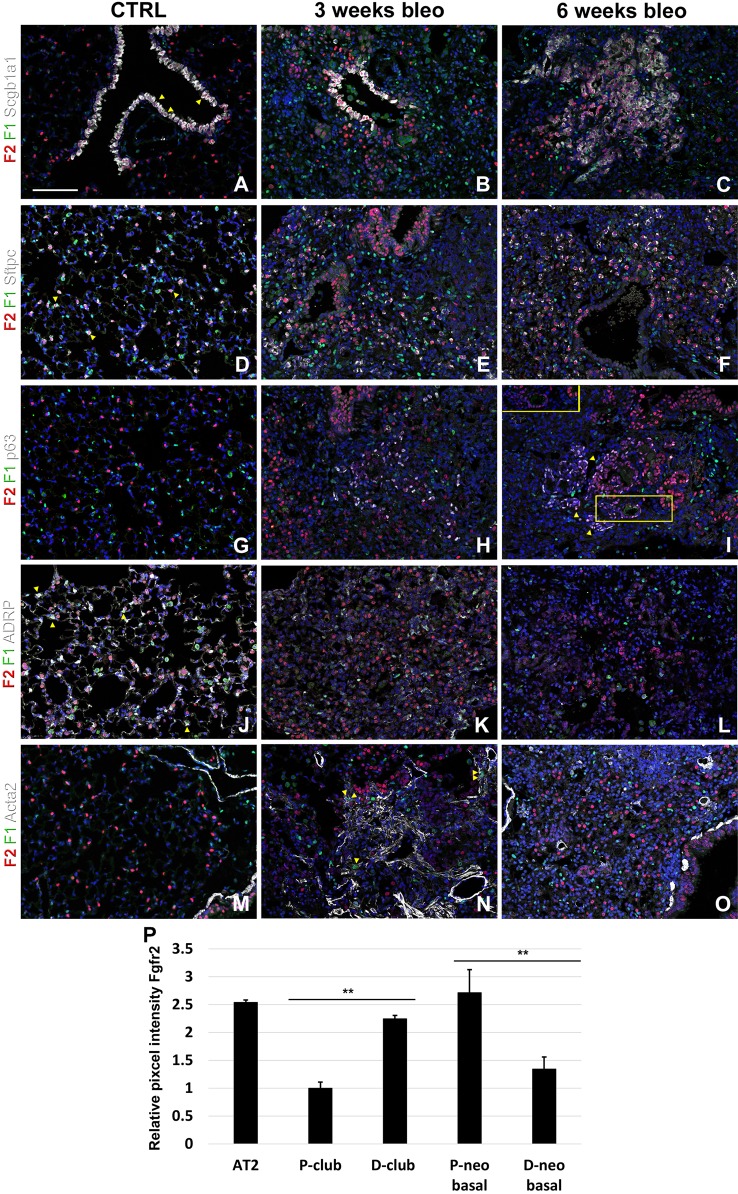
Fgfr1 and Fgfr2 expression in the adult lung after bleomycin injury. **(A–X)** Immunostaining on ctrl and naphthalene injured adult *Fgfr1-Cerulean;Fgfr2-mCherry* lungs for GFP (Fgfr1-Cerulean, green), RFP (Fgfr2-mCherry, red), Scgb1a1 (white), Sftpc (white), p63 (white), ADRP (white), or Acta2 (white) (arrowheads indicate double labeled cells whereas frames show only GFP and RFP). **(P)** Quantification of relative average pixel intensity for Fgfr2 in alveolar type 2 (AT2) cells and proximal and distal club vs neo-basal cells after bleomycin injury. Data are mean ± s.e.m. **P < 0.01, as determined by a two-tailed t-test; n ≥ 10 biological replicates for each experimental group. Scale bars, 100 µm.

Lastly, we monitored the expression of Fgfr1 and Fgfr2 upon bleomycin injury. We found Fgfr2 to be higher in the distal airway club cells compared to proximal airway club cells at 3 and 6 weeks after bleomycin injury (**Figures 6A–C, P**). These findings are consistent with our previous report on the role of Fgf10-Fgfr2 signaling in alveolar epithelial regeneration by bronchial epithelial stem cells (Yuan et al., 2019). We found similarly high expression of Fgfr2 in regeneration AT2 cells upon bleomycin injury (**Figures 6D–F, P** and **S2**) and in proximal neo-basal cells but significantly lower expression of Fgfr2 in more distal neo-basal cells (**Figures 6G–I, P**), consistent with our previous finding that Fgfr2 is required for the development of neo-basal cells upon bleomycin injury and that increased Fgf10 signaling can drive these cells along the AT2 cell lineage whereas reduced Fgfr2 signaling leads to their differentiation into AT1 cells (Yuan et al., 2019). Interestingly, we found Fgfr1 expression in myofibroblasts upon bleomycin injury (**Figures 6J–O**) consistent with them being derived from lipofibroblasts (El Agha et al., 2017; Yuan et al., 2019).

## Discussion

We found that during early lung development, Fgfr1 and Fgfr2 show different expression patterns. Fgfr1 is more dominantly expressed in mesenchymal cells; whereas, Fgfr2 is preferentially expressed in epithelial cell lineages (**Figures 1**–**3**). This trend continuous during the postnatal stage, with strong Fgfr2 expression in AT2 and basal cells, lower expression in club cells, and strong Fgfr1 expression in mesenchymal alveolar niche lipofibroblast cells and vascular smooth muscle cells (**Figure 4**). In the developing lung, Fgf10, expressed and released by the distal mesenchyme, binds and activates Fgfr2 on distal tip epithelial progenitors epithelium to keep them in a progenitor-like state by inducing Sox9 (Volckaert et al., 2013b; Volckaert et al., 2019). In this study, we detected that at E15.5, Fgfr2 expression becomes downregulated in the Sox9^+^ distal epithelium (**Figures 1B, G**) and upregulated more proximally, indicating that downregulation of Fgfr2 signaling distally is required for the lung to transition from a branching program into an alveolar differentiation program.

After naphthalene injury, Fgf10 signaling is reactivated in the parabronchial smooth muscle cells (PSMCs), and Fgfr2 is upregulated in neuroendocrine bodies, suggesting that Fgf10 signaling may activate this reserve stem cell population upon injury (**Figures 5M–P**), consistent with previous findings (Volckaert et al., 2011; Volckaert et al., 2013a; Lee et al., 2017).

We recently discovered that Fgf10-Fgfr2b signaling is critical for the neo-basal cell generation and alveolar epithelial regeneration after bleomycin injury (Yuan et al., 2019). From this study, we find similarly high levels of Fgfr2 in distal airway club cells, AT2 cells, and proximal-neo basal cells supporting our previous findings. Intriguingly, lineage tracing data from our previous study indicate that the majority of neo-basal cells, which appear near BADJs after injury, are generated through the dedifferentiation of club cells and function as a transitional epithelial cell lineage to assist in alveolar epithelial regeneration (Yuan et al., 2019). The dynamic reduction in Fgfr2 expression in a subset of p63^+^ basal cells (**Figures 6H, I, P**) implies their direct differentiation into AT1 cells, whereas increasing Fgfr2 expression might help boost basal cell to AT2 cell reprogramming (Yuan et al., 2019). The differentiation of lipofibroblasts (LIFs) into myofibroblast (MYFs) is a classical feature during bleomycin injury (Yuan et al., 2018). Fgf10, as one of the most important fibroblast growth factors during lung development binds with high affinity to Fgfr2b but has a lower affinity to Fgfr1b. During homeostasis, in adult lungs, Fgf10 is expressed in the mesenchymal niches between the cartilage rings where normally basal cells reside in the trachea and in the lipofibroblast or mesenchymal alveolar niche cells adjacent to AT2 cells in the alveoli. Whole lung *Fgf10* expression goes up after bleomycin injury due to the amplification of myofibroblasts yet on a per cell basis Fgf10 expression is reduced in myofibroblasts compared to the lipofibroblasts they are derived from (Yuan et al., 2019). It is possible that Fgf10 expressing mesenchymal alveolar niche lipofibroblasts express Fgfr1b and therefore, respond to Fgf10 signaling in an autocrine fashion. Future experiments will need to be designed to assess this (Al Alam et al., 2015).

## Materials and Methods

### Contact for Reagent and Resource Sharing

Further information and requests for resources and reagents should be directed to and will be fulfilled by the Lead contact, SL (sdelanghe@uabmc.edu).

### Experimental Model Details

All mice were bred and maintained in a pathogen-free environment with free access to food and water. Both male and female mice were used for all experiments. *Fgfr1^Cerulean^* (JAX 030708), *Fgfr2^mCherry^* (JAX 030710) (Molotkov et al., 2017) mice were obtained from Jackson laboratories and crossbred to homozygosity. For bleomycin injury, adult 8 week old mice were intratracheally instilled with 50 µl bleomycin (1U/kg body weight for females, and 0.8U/kg body weight for males) as previously described (Yuan et al., 2019). For naphthalene injury, adult 8 week old mice were intraperitoneally injected with naphthalene dissolved in corn oil (325 mg/kg body weight for males and 300 mg/kg body weight for females) as previously described (Volckaert et al., 2011). All experiments were approved by the University of Alabama at Birmingham Institutional animal care and use committee.

### Immunohistochemistry and Fluorescence

All staining was done on paraffin sections of formalin-fixed lungs or tracheas. Immunofluorescent staining was performed with the following primary antibodies: goat anti-Scgb1a1 (1:200; clone T-18; sc-9772; Santa Cruz Biotechnology Inc.), rabbit anti-Scgb1a1 (1:500; WRAB-CCSP; Seven Hills Bioreagents), mouse anti-α-Actin (1:500; clone 1A4; sc-32251; Santa Cruz Biotechnology Inc.), chicken anti-GFP (1:250; GFP-1020; Aves Labs Inc.), rabbit anti-Keratin 5 (1:200; clone EP1601Y; MA5-14473; Thermo Fisher Scientific), chicken anti-Keratin 5 (1:500; clone Poly9059; 905904; BioLegend); rabbit anti-p63 (ΔN) (1:500; clone poly6190, 619002; BioLegend), 3862S; mouse anti-p63 (1:50; clone 4A4; CM163B; Biocare Medical), rabbit anti-RFP (1:200; 600-401-379; Rockland Immunochemicals Inc), mouse anti-RFP (1:200; sc-390909; Santa Cruz Biotechnology Inc.), rabbit anti-Sftpc (1:200; WRAB-9337; Seven hills bioreagents), mouse anti-Hop (1:100; Clone E-1; sc-398703; Santa Cruz Biotechnology Inc.), guinea pig anti-ADRP (Adipophilin) (1:200; 20R-AP002; Fitzgerald Industries), rabbit anti-Sox2 (1:1,000; WRAB-1236; Seven hills bioreagents), goat anti-Sox9 (1:500; AF3075; R&D systems), rabbit anti-CGRP (1:5,000; C8198; Sigma) and Mouse anti-Foxj1 (1:500, 14-9965-82, Invitrogen).

After deparaffinization, slides were rehydrated through a series of decreasing ethanol concentrations and antigens unmasked by either microwaving in citrate-based antigen unmasking solution (Vector Labs, H-3000) or by incubating sections with proteinase K (7.5 μg/ml) (Invitrogen, 25530-049) for 7 min at 37°C. Tissue sections were then washed in TBS with 0.1% Tween-20 and blocked with 3% bovine serum albumin (BSA), 0.4% Triton X-100 in Tris buffered saline (TBS) for 30 min at room temperature followed by overnight incubation of primary antibodies diluted in 3% BSA, 0.1% Triton X-100 in TBS. The next day, slides were washed in TBS with 0.1% Tween-20 and incubated with secondary antibodies diluted in 3% BSA, 0.1% Triton X-100 in TBS for 3h at room temperature. All fluorescent staining was performed with appropriate secondary antibodies from Jackson Immunoresearch, except for mouse anti-Hop (1:500; A-21125; Thermo Fisher Scientific). Slides were mounted using Vectashield with (Vector Labs, H-1200) or without DAPI (Vector Labs, H-1000) depending on immunostaining.

### Microscopy and Imaging

Tissue was imaged using a micrometer slide calibrated Zeiss LSM800 Laser scanning confocal microscope using ZEN imaging software. Images were processed and analyzed using Zen blue and Adobe Photoshop software. Average immunostaining intensity was quantified after segmentation and thresholding for pixel intensity.

## Data Availability Statement

All datasets generated for this study are included in the article/**Supplementary Material**.

## Ethics Statement

All experiments were approved by the University of Alabama at Birmingham Institutional Animal Care and Use Committee.

## Author Contributions

TY designed and performed experiments, analyzed data, and wrote and edited the manuscript. KK performed experiments, analyzed data, and edited the manuscript. HL, JY, SG, and SH performed experiments. JZ edited the manuscript. SDL conceived and led the project, performed experiments, analyzed data, and wrote and edited the manuscript.

## Funding

This study was supported by NIH R01 HL126732, HL132156, and HL146160 awards to SDL; and Cystic Fibrosis Foundation (CFF) YUAN19F0 awards to TY.

## Conflict of Interest

The authors declare that the research was conducted in the absence of any commercial or financial relationships that could be construed as a potential conflict of interest.

## Supplementary Material

Figure S1 Fgfr1, Fgfr2 and Scgb1a1 expression after naphthalene injury. Immunostaining on ctrl and naphthalene injured adult *Fgfr1-Cerulean;Fgfr2-mCherry* lungs for GFP (Fgfr1-Cerulean, green), RFP (Fgfr2-mCherry, red), Scgb1a1 (white).

Figure S2 Fgfr1, Fgfr2 and Sftpc expression after bleomycin injury. Immunostaining on ctrl and bleomycin injured adult *Fgfr1-Cerulean;Fgfr2-mCherry* lungs for GFP (Fgfr1-Cerulean, green), RFP (Fgfr2-mCherry, red), Sftpc (white).
