# Supplementary figures and images for: Temporospatial Expression of Fgfr1 and 2 During Lung Development, Homeostasis, and Regeneration
Source: Front Pharmacol. 2020 Mar 2;11:120. doi: 10.3389/fphar.2020.00120 (PMC7061767; doi:10.3389/fphar.2020.00120)

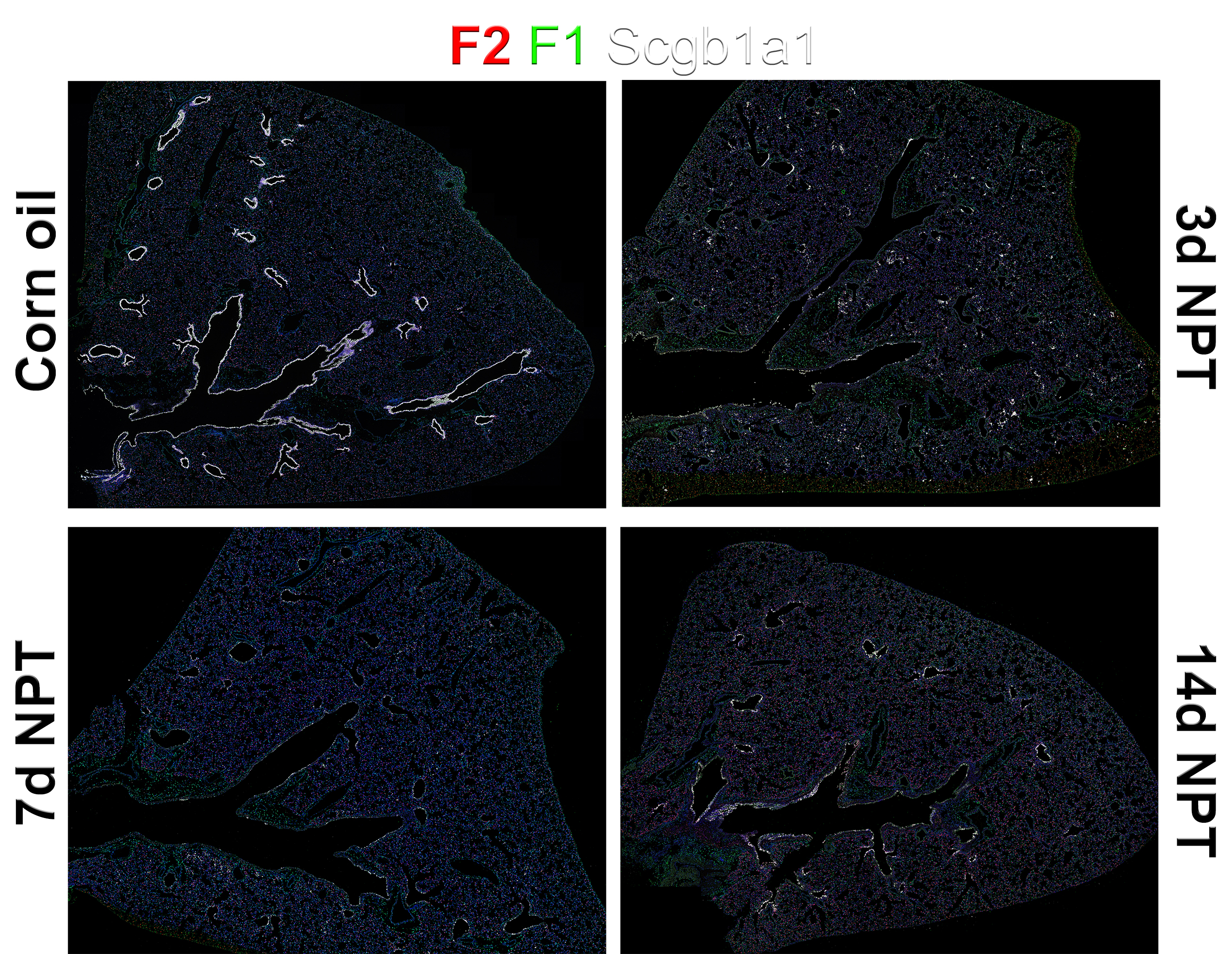

Supplement: Figure S1 — Fgfr1, Fgfr2 and Scgb1a1 expression after naphthalene injury. Immunostaining on ctrl and naphthalene injured adult Fgfr1-Cerulean;Fgfr2-mCherry lungs for GFP (Fgfr1-Cerulean, green), RFP (Fgfr2-mCherry, red), Scgb1a1 (white). [file Image_1.jpeg]

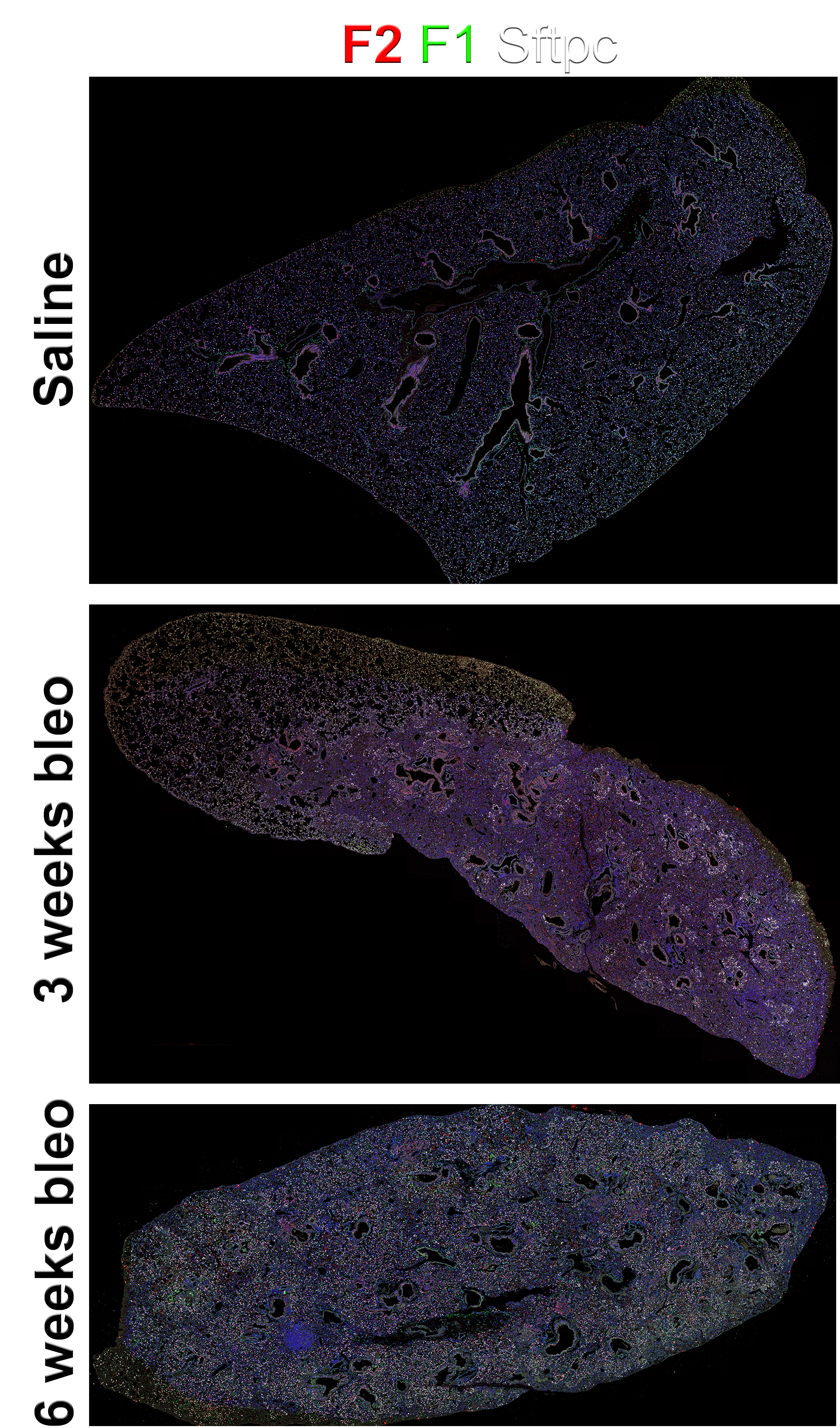

Supplement: Figure S2 — Fgfr1, Fgfr2 and Sftpc expression after bleomycin injury. Immunostaining on ctrl and bleomycin injured adult Fgfr1-Cerulean;Fgfr2-mCherry lungs for GFP (Fgfr1-Cerulean, green), RFP (Fgfr2-mCherry, red), Sftpc (white). [file Image_2.jpeg]
